# Differential diagnosis between syndrome of inappropriate antidiuretic hormone secretion and cerebral/renal salt wasting syndrome in children over 1 year: proposal for a simple algorithm

**DOI:** 10.1007/s00467-021-05250-1

**Published:** 2021-09-01

**Authors:** Flaminia Bardanzellu, Maria Antonietta Marcialis, Roberta Frassetto, Alice Melis, Vassilios Fanos

**Affiliations:** Neonatal Intensive Care Unit, Department of Surgical Sciences, AOU and University of Cagliari, SS 554 km 4, 500, 09042 Monserrato, Italy

**Keywords:** Hyponatremia, Hypouricemia, Children, Kidney disease, Fractional urate excretion

## Abstract

Hyponatremia, especially if acute and severe, can be a life-threatening condition. Several conditions can trigger hyponatremia. In this review, we will discuss two conditions that can determine euvolemic hyponatremia: the cerebral/renal salt wasting (CRSW) syndrome and the syndrome of inappropriate secretion of antidiuretic hormone (SIADH), including the two subtypes: reset osmostat (RO) and nephrogenic syndrome of inappropriate antidiuresis (NSIAD) and their differential diagnoses. Despite the passage of over 70 years since its first description, to date, the true etiopathogenesis of CRSW syndrome, a rare cause of hypovolemic/euvolemic hyponatremia, is almost unknown. SIADH, including RO and NSIAD, is sometimes difficult to differentiate from CRSW syndrome; in its differential diagnosis, the clinical approach based on the evaluation of the extracellular volume (ECV) was proven insufficient. We therefore suggest a simple diagnostic algorithm based on the assessment of the degree of hyponatremia, urinary osmolality, and the assessment of the fraction of urate excretion (FEUa) in conditions of hyponatremia and after serum sodium correction, to be applied in children over 1 year of life.

## Introduction

Hyponatremia is a fluid balance disorder defined as serum sodium levels less than 133–135 mEq/L. In mild hyponatremia, serum sodium levels are ≥ 130 mEq/L; in moderate cases, they are 120–130 mEq/L; while in severe cases, serum sodium levels are less than 120 mEq/L [1]. Hyponatremia-associated consequences have been reported in cases of serum levels < 125 mEq/L [2]. In the case of severe hyponatremia, mortality rapidly increases, especially if acute [1].

Hyponatremia can be quite frequent in both children and newborns. Among preterm newborns, the exact incidence is difficult to estimate and it seems to affect two-thirds of very low-birth weight (VLBW) infants [3].

Many types of classification are useful in defining the extent and type of hyponatremia. It can be defined as acute until 48 h of onset, while chronic hyponatremia is a well-established condition which lasts for almost 48 h [1].

Hyponatremia can be also defined and characterized, according to plasma osmolality, in hysotonic hyponatremia (280–295 mOsm/L), hypertonic hyponatremia (> 295 mOsm/L), and hypotonic hyponatremia (< 280 mOsm/L). Moreover, hyponatremia can be divided into subtypes according to extracellular volume (ECV) status, represented by the intravascular compartment, which can be reduced (hypovolemic hyponatremia), normal (euvolemic hyponatremia), and increased (hypervolemic hyponatremia) [1]. A complete description of hyponatremia, its potential causes, differential diagnoses, and therapeutic options for a correct management were subjects of our previous reviews [1, 3] and are beyond the scope of this discussion.

The aim of this paper is the discussion of two rare causes of hyponatremia: cerebral/renal salt wasting (CRSW) syndrome and syndrome of inappropriate antidiuretic hormone (SIADH) synthesis (including its subtypes reset osmostat (RO) and nephrogenic syndrome of inappropriate antidiuresis (NSIAD)), which can both represent hypotonic euvolemic conditions in which the differential diagnosis can be a difficult challenge, since the only evaluation of ECV can be insufficient. We therefore propose a simple diagnostic algorithm based on the assessment of hyponatremia, urinary osmolality, and the assessment of the fraction of urate excretion (FEUa) in conditions of hyponatremia and after serum sodium correction, to be applied in children over 1 year of life.

Considering that there is sufficient evidence on the values of FEUa in children (more than 1 year old) [4], we could consider the importance of this diagnostic tool from this age onward. In newborns, unfortunately, FEUa is difficult to quantify, especially if premature. “Normal” values are wide and poorly established; the articles on the topic are old and not conclusive [5, 6]. Therefore, we decided to exclude newborns from our algorithm.

Hypouricemia is characterized by plasma uric acid levels less than or equal to 2 mg/dl in adults [7]. There may be various conditions or pathologies underlying this condition, and practically, the first step for a correct differential diagnosis is FEUa determination. In fact, hypouricemia associated with reduced FEUa is determined by defects in the production of uric acid (including xanthinuria, treatment with allopurinol or rasburicase, neoplasms, liver function abnormalities, or glomerular hyperfiltration), while in the case of increased FEUa, the cause can be a defect involving the kidney proximal tubular transport of uric acid (including isolated or complex tubulopathies, the administration of salicylates, neoplasms, cirrhosis, diabetes mellitus, and SIADH), as extensively reviewed [7].

In this review, we will use the acronym AVP (arginine vasopressin) instead of ADH (antidiuretic hormone), which are the same.

Urinary urate represents about 70% of the total daily production, while the remainder undergoes fecal elimination; moreover, urinary uric acid is about 10% of the filtered amount [7].

Four phases have been identified to explain the kidney uric acid excretion. Circulating uric acid undergoes passive glomerular filtration; a fraction is reabsorbed in the proximal tubule; then, uric acid can be secreted in the tubular lumen [7, 8]. Finally, a postsecretory reabsorption, distal from secretion, can occur [7, 9]. All these phases occur in the glomerulus and in the proximal tubule.

Thus, hypouricemia can represent a biochemical marker for primary or secondary tubulopathies, and the defect underlying the increased excretion of uric acid may depend on isolated or combined impaired presecretory and/or postsecretory reabsorption mechanisms or by an increased tubular secretion [7].

Several tubular uric acid transporters, intracellular carriers, and related genes have been identified to date, including some urate-sodium co-transporters; thus, since sodium reabsorption is coupled to that of uric acid, through the steps specified above, urinary loss of sodium is associated with that of uric acid [10].

In SIADH, excessive tubular water reabsorption determines the expansion of the intravascular, extracellular, and intracellular volume and leads to hemodilution, with consequent hyponatremia and hypouricemia. This is similar to what occurs in situations of increased fluid intake such as in polydipsia or in cases of increased intravenous fluid intake. SIADH hyponatremia with concomitant hypouricemia can be corrected by fluid restriction, different from what happens in CRSW syndrome, in which hypouricemia persists despite serum sodium correction [7].

**Fig. 1:**
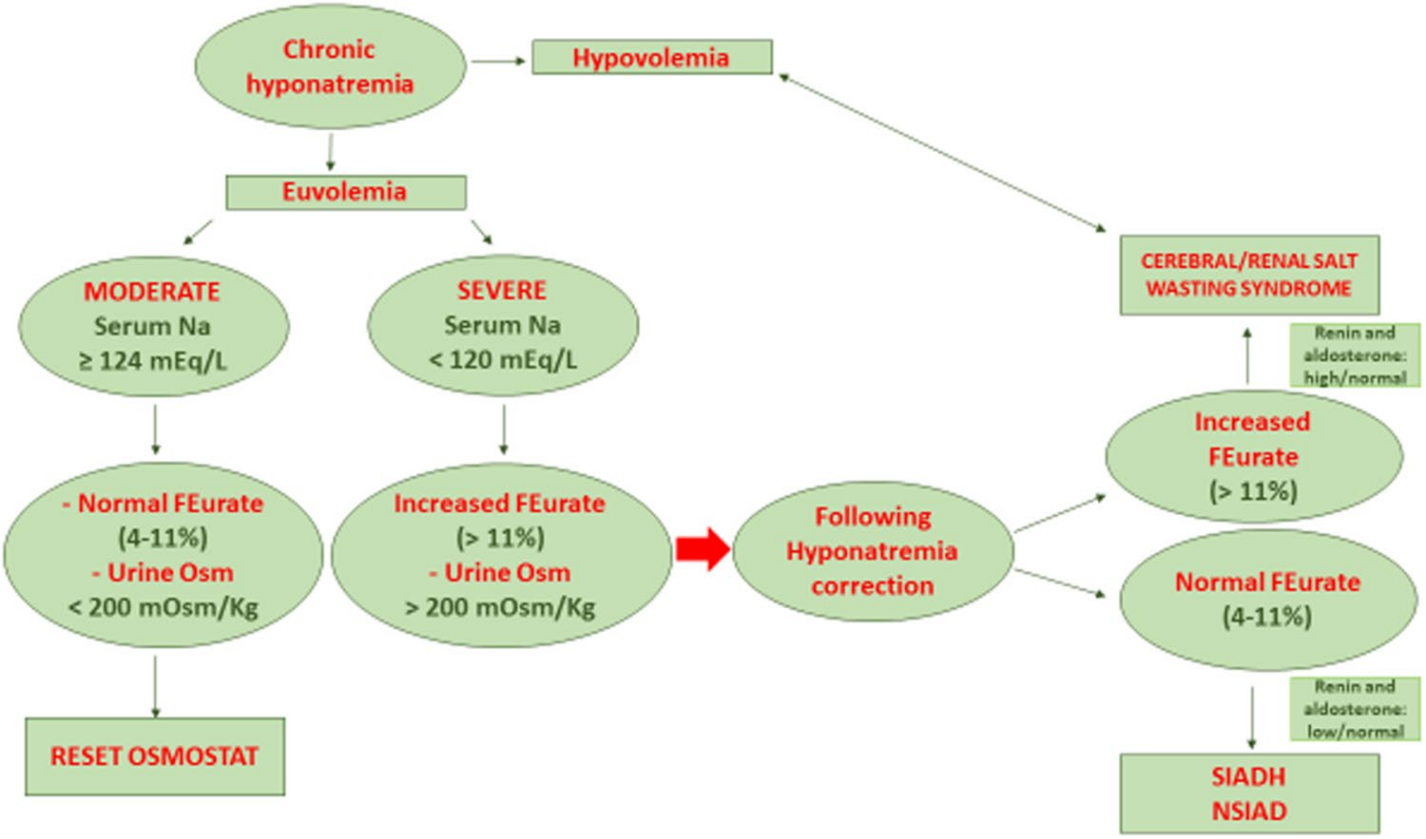
Extremely rare causes of chronic hyponatremia. Diagnostic algorithm for children over 1 year of life

In Fig. 1, we report the extremely rare causes of chronic hyponatremia with a diagnostic algorithm based on FEUa. In Table 1, the main laboratory differences among CRSW syndrome, SIADH, NSIAD, and RO are reported.

**Table 1:** Extremely rare causes of chronic hyponatremia and diagnostic criteria in children over 1 year of life

	FEUa	Serum hyponatremia	Urine osmolarity after isotonic saline infusion (mOsm/L)	AVP	Renin and aldosterone
Cerebral/renal salt wasting syndrome	Increased	Severe	< 100	Increased	High/normal
SIADH	Increased	Severe	> 100	Increased	Low/normal
NSIAD	Increased	Severe	> 100	Low/undetectable	Low/normal

## Cerebral/renal salt wasting syndrome

### Etiology and epidemiology in adults

CRSW syndrome is a rare and still not completely understood disorder. It is characterized by a kidney loss of sodium, leading to polyuria, intravascular volume contraction, and hyponatremia, with increased AVP levels, because of the baroreceptor-mediated stimulus. The first three cases were described in 1950 by Peters et al. [11], and 7 years later, the first report of SIADH was published [12].

The affected patients were all carriers of cerebral disease (intracranial hemorrhage, bulbar poliomyelitis, and meningitis) and showed, in addition to hyponatremia, concentrated urine, high urine sodium concentration, and intravascular volume depletion [11].

Later, Cort [13] reported another similar case and named the condition as cerebral salt wasting (CSW) syndrome, because the four cases reported to date were all associated with cerebral diseases. In adults, subarachnoid hemorrhage is one of the most common CSW syndrome triggers; other causes can be represented by brain injuries, stroke, neurosurgical interventions, intracranial tumors, sepsis, and viral and bacterial meningitis [14–17].

The incidence and prevalence of CSW syndrome is difficult to define among adults; however, CSW syndrome underlies at least 25% of cases of severe hyponatremia after aneurysmal subarachnoid hemorrhage [18].

Following the first CSW syndrome cases, it was observed that polyuria, intravascular volume contraction, hyponatremia, and increased AVP levels could also occur in patients without cerebral diseases; thus, the acronym CSW can be inappropriate and it has been proposed to change it to renal salt wasting (RSW) syndrome [19]. Consequently, we preferred to adopt the term cerebral/renal salt wasting (CRSW) syndrome, suggested by Maesaka et al. [20].

Although the exact pathogenic mechanism underlying natriuresis in CRSW syndrome is unclear, in some cases, it could be caused by the release of vasodilator mediators, such as atrial natriuretic peptide (ANP), brain natriuretic peptides (BNPs), and C-type natriuretic peptide (CNP), that can promote diuresis, natriuresis, and hyponatremia. Although BNP has a major cardiac origin, it can also be produced by the hypothalamus, undergoing release during pathological processes [21].

According to the hypotheses of other authors, a direct injury that affects the central nervous system, resulting in a reduced stimulation of the proximal kidney tubules, may cause abnormal natriuresis, diuresis, and hyponatremia [22, 23]. Natriuresis is followed by volume depletion, secondary AVP increase, and hyponatremia. In cases of subarachnoid hemorrhage, CRSW syndrome may represent a protective measure which limits intracranial pressure increase [24]. In addition, natriuresis can also be due to a defect in sodium reabsorption in the proximal tubule, accompanied by increased uric acid and urea excretion rates occurring in CRSW [25].

### CRSW syndrome in children

CRSW syndrome is a not fully defined cause of secondary hyponatremia among children, and numerous conditions have been identified as possible triggers of this disorder [26]. During a 5-year study period, Bussmann et al. [27] observed that 9 out of 195 pediatric patients (4.6%) with acute CNS disease also presented CRSW syndrome, with hyponatremia persisting up to 3 days.

In 2006, von Bismarck et al. [26] identified nine CRSW syndrome–affected patients, all affected by brain diseases. The onset of CRSW syndrome occurred within 2–3 days after the trauma or neurosurgery intervention and lasted up to 22 months [26]. In a group of 110 pediatric patients (up to 18 years old), CRSW syndrome appeared to be more frequent in children younger than 3 years; among them, males were more affected than females (63% vs. 37%). In this population, the typical CRSW syndrome onset was within the first 8 days after the cerebral insult [28]. Generally, CRSW syndrome lasts a few weeks or months, or sometimes longer, after onset [18].

In a retrospective study of 159 pediatric patients affected by suprasellar tumors, 6 (3.6%) cases of persistent CRSW syndrome were described. Among them, the electrolytic disorder resolved in a month in one patient and in a year in another case, while in 4 patients, salt supplement was still needed after 33–43 months [29].

Similar to what happens in adults, CRSW syndrome in children mostly represents a consequence of intracranial surgery, subarachnoid hemorrhage, meningoencephalitis (most frequently of tuberculous etiology), and brain injuries and is frequently described in sporadic case reports. CRSW syndrome can also occur after ketamine infusion or can be associated with medulloblastoma, status epilepticus, Kawasaki disease, hematopoietic stem cell transplantation, or lissencephaly [28–35].

### CRSW syndrome diagnosis

In 2006, and again in 2013, the Expert Panel Recommendations of Hyponatremia included CSW syndrome among the causes of hypovolemic hyponatremia. Since the assessment of the intravascular volume is rather complicated, the experts suggested using indirect methods such as an accurate medical history, physical examination, and laboratory investigation to assess volume depletion typical of CRSW syndrome. They recommended considering hypotension, increased pulse rate, weight loss, negative fluid balance, dry mucus membranes, and decreased skin turgor just before and during the first period of hyponatremia as signs of volume depletion and dehydration. In addition, the authors emphasized that blood urea nitrogen (BUN), hematocrit and serum creatinine are generally increased in CSW syndrome, uric acid, and serum osmolality are decreased, and urine sodium excretion and urine osmolality are increased [25, 36]. CSW syndrome, following an early hypovolemic phase, can become euvolemic: this depends on AVP increase secondary to hypovolemia [20, 36, 37].

Considering the difficulty in distinguishing extracellular hypovolemia from euvolemia, in suspicious cases, the authors suggested performing a test infusion with isotonic saline solution, which determines a sustained increase in sodium levels in the case of CRSW syndrome. Unfortunately, this overload test can be harmful, especially if performed in the neonatal period [25, 36].

## Syndrome of inappropriate antidiuretic hormone secretion

### Etiology and clinical features

SIADH is due to the unsuppressed release of AVP from the pituitary gland, or even non-pituitary sources, and its excessive action on vasopressin kidney receptor V2R [38]. The first description of such a condition was done in 1957 regarding two patients affected by lung cancer, by Bartter and Schwartz [39]. SIADH is characterized by reduced water excretion, resulting in hyponatremia, euvolemia, plasma hypoosmolality, and inappropriately concentrated urine, with increased urinary osmolality, increased natriuresis, and increased AVP circulating levels, in cases of normal kidney, heart, liver, adrenal, and thyroid function [40, 41]. Moreover, FEUa is increased (> 11%) and normalizes (4–11%) after serum sodium correction [42]. Such condition is reversible following water restriction [40]. AVP increase, typical in SIADH [43, 44], is defined as inappropriate, since it occurs without an adequate trigger (osmotic or hemodynamic) [40].

Numerous causes have been associated with SIADH, including cerebral disorders (i.e., stroke, hemorrhage, infection, trauma, mental illness, and psychosis), increasing AVP pituitary release, tumors (more frequently, small cell lung cancer, but even extrapulmonary malignancies can cause ectopic AVP secretion), other pulmonary diseases, several drugs, surgical interventions, general anesthesia, infections such as human immunodeficiency virus (HIV) infection, stress, and many others [38, 40].

To date, four types of SIADH have been described. In type A, AVP secretion is unregulated. A basal AVP secretion despite normal regulation by osmolality characterizes type B. Type C is characterized by a “reset osmostat” (RO) and is discussed as an autonomous entity later in this review. Finally, type D is the kidney genetic form, NSIAD, discussed elsewhere [45].

The incidence of SIADH is difficult to estimate, since it is a transient condition associated with different etiologies. Although SIADH incidence was reported to increase with age, it seems to represent a cause of hyponatremia more frequent than expected in children; most cases are acute and transient disorders, especially in patients hospitalized for respiratory or cerebral infections, or during post-intervention periods [38, 40].

### SIADH: diagnosis and treatment

Hyponatremia with euvolemia should arouse the clinical suspicion of SIADH. Moreover, the clinical criteria formulated by Bartter and Schwartz [39] can help in the diagnosis and are represented by serum sodium less than 135 mEq/L, serum osmolality less than 275 mOsm/kg, natriuria greater than 40 mEq/L, urine osmolality greater than 100 mOsm/kg, euvolemia (no edemas, no volume depletion, normal blood pressure), absence of other causes of hyponatremia (adrenal insufficiency, hypothyroidism, heart failure, pituitary insufficiency, kidney disease with salt wastage, liver disease, drugs that impair kidney water excretion), and hyponatremia by fluid restriction correction [39].

In SIADH, water reabsorption primarily determines an increase in intracellular water, and only a low and transient increase in ECV (intravascular space) in its early stages (with a transient weight increase). For this reason, patients are not edematous and this type of hyponatremia is defined as euvolemic [46].

In SIADH, hyponatremia represents the most concerning manifestation, especially if acute, due to the risk of cerebral edema and seizures, potential permanent cerebral damage, and even death. Thus, acute hyponatremia should be corrected, while avoiding a too-rapid rise in sodium serum levels. In chronic hyponatremia, due to SIADH, adaptive brain mechanisms make hyponatremia better tolerated by the brain and require very slow correction [1, 3, 38].

However, the adequate treatment strictly depends on the associated condition; the accurate evaluation of children with hyponatremia can orient its correct management [40].

Water restriction is a key point in treatment [38], and furosemide can be useful [40]. Regarding specific SIADH treatment, oral urea, an osmotic diuretic drug, is recommended by the European Clinical Practice Guidelines for SIADH-induced hyponatremia [47, 48].

In the kidney, urea can freely pass through the glomerulus and is eliminated together with free water. Moreover, it can reduce intracranial and intraocular pressure [47].

The class of vaptans, V2R antagonists, constitutes another reliable treatment for hyponatremia, efficacious in SIADH (but not in NSIAD and RO). Vaptans reversibly block the AVP link to V2R, promoting aquaresis and therefore serum sodium normalization [49].

Tolvaptan and conivaptan, according to the US Food and Drug Administration (FDA), can be used for short periods in SIADH in adult patients [50–52], although their safety profile still requires confirmation [53] and some side effects can be observed, including thirst, dry mouth, nausea, polyuria, urinary tract infections, hypotension, fever, hyperkalemia, asthenia, headache, and excessive but reversible sodium correction [49, 50].

These drugs have been evaluated only after the resolution of severe hyponatremia [50]; moreover, little evidence is available on vaptan use in children [54]. Only three children affected by SIADH were treated, and despite good results, the drugs have not been approved for pediatric patients [55]. In patients with SIADH, the prognosis depends on the underlying condition, often being poor in cases of malignancies [40].

### CRSW syndrome and SIADH: similarities and differences

CRSW syndrome and SIADH both show some common findings, including hyponatremia; serum osmolality less than 275 mOsm/kg; normal kidney, adrenal, and thyroid functions; potential association with cerebral diseases; decreased serum uric acid level; increased sodium excretion (greater than or equal to 20–30 mEq/L); and increased urine osmolality (> 100–200 mOsm/kg). Natriuresis is one of the cardinal criteria of SIADH diagnosis [39] and is caused by the increase in sodium glomerular filtration rate, rather than a higher urinary output of sodium, which occurs in CSW syndrome [18]. Furthermore, as discussed below, AVP levels could be increased in both conditions and patients can show euvolemia (without edema or dehydration). Recent evidence showed that volume evaluation can fail in the differentiation of CRSW syndrome and SIADH [20, 37].

In fact, CRSW and SIADH are both forms of hypotonic hyponatremia, being characterized by low plasma osmolality. These conditions can be euvolemic (without an increase in the intravascular fluid compartment which is part of the ECV), such as SIADH, NSIAD, and RO or hypovolemic (characterized by a reduction involving intravascular fluid), such as CRSW.

However, CSW syndrome, initially hypovolemic, can successively develop euvolemia; this is due to compensatory mechanisms of AVP release secondary to hypovolemia, despite hyponatremia, because the hypovolemic stimulus is more life-saving. Thus, ECV is not conclusive in the diagnosis of these conditions [20, 36, 37].

In comparison, FEUa allows the differentiation between CRSW syndrome and SIADH with excellent specificity, although serum sodium correction is required to indicate the different uric acid excretions. The normal FEUa value is between 4 and 11%. Uric acid is exclusively excreted in the kidney proximal tube. Both SIADH and CRSW syndrome show hypouricemia with increased FEUa (> 11%). Hypouricemia following extracellular volume expansion is associated with decreased reabsorption of both sodium and uric acid; therefore, in SIADH, water restriction returns serum sodium, uricemia, and FEUa to physiologic values. Conversely, a persistently elevated FEUa (FEUa > 11%) after hyponatremia correction with salt and water identifies the condition as CRSW syndrome [7, 20].

Because of volume depletion, plasma renin and aldosterone levels are generally elevated in CRSW syndrome. On the contrary, in SIADH, their values are low-normal [19]. Such a difference, as illustrated in Fig. 1 and Table 1, can be useful for the differential diagnosis. However, compensatory phases may also occur in which renin and aldosterone may be normal in the two syndromes. Therefore, it would be correct to state that elevated renin and aldosterone findings are quite specific for CRSW, which, however, cannot be excluded in case of normal values [56].

A challenge test through the infusion of a saline solution (0.9%), although unsafe and not recommended in newborns and children, could be useful to distinguish CRSW and SIADH. In the first condition, the blood volume normalizes, AVP reduces, and serum sodium undergoes normalization, since the kidney is able to eliminate a water load. In SIADH, due to the high AVP secretion, water is retained, serum sodium is reduced, and urinary sodium is increased. This is reported in Table 1.

Drugs like fludrocortisone can successfully restore fluid volume status in CRSW syndrome, enhancing sodium and water reabsorption from the distal tubules [57].

In conclusion, the determination of serum uric acid and FEUa can distinguish CRSW syndrome from SIADH, despite serum sodium level correction necessary for the differential diagnosis, and is safe even in young patients.

## Subtypes of SIADH: reset osmostat

The osmostat is the hypothalamic center of osmolality regulation. In physiological conditions, plasma osmolality is regulated between 275 and 295 mOsm/kg; it occurs through AVP secretions, which is stimulated when plasma osmolality increases, while it is suppressed when osmolality decreases below 280 mOsm/kg. RO is a rare condition described in 1976 and classified among the third type of SIADH (type C SIADH). Although generally characterized by hyponatremia, RO has also been described in hypernatremic patients who suffered from hypothalamic and pituitary lesions [58]. The classical form of the disorder, associated with hyponatremia, is related to a change in the normal plasma osmolality threshold, in which AVP secretion still occurs with sodium plasma levels of 125–130 mm/L and plasma osmolality between 250 and 275 mOsm/kg. RO is commonly associated with hypouricemia; normal FEUa (4–11%) euvolemia; normal adrenal, kidney, and thyroid functions; and decreased plasma osmolality despite a conserved kidney ability to dilute and concentrate urine in response to AVP. Affected patients show a constitutive AVP secretion, despite plasma hypotonicity, with a subsequent increased water reabsorption in the kidney, leading to hyponatremia, refractory to salt overload. RO patients present diluted urine with urine osmolality lower than plasma osmolality [59].

RO can cause mild-moderate hyponatremia. Serum sodium (125–135 mmol/L) usually remains stable despite variations in water and sodium intake; therefore, RO does not respond to oral sodium supplementation or to fluid restriction. Moreover, fludrocortisone is ineffective in treating RO [42]. Consequently, serum sodium correction is not necessary [60].

### Reset osmostat: etiology and epidemiology in adults

According to the literature, about 36% of adult patients with SIADH are affected by the subtype RO. RO has been reported in pregnant women showing serum osmolality 5–10 mOsm/kg lower than normal values and serum sodium concentration 4–5 mmol/L lower than normal [59, 61, 62]. Although RO etiology is unknown, the disorder could be due to a change of osmoreceptor cell metabolism [58]. It has been associated with several conditions, including infections (tuberculosis, encephalitis pneumocystis pneumonia), carcinomas, quadriplegia, cerebral hemorrhage, dementia (Lewy bodies), psychosis, alcoholism, and elderly. In RO, hyponatremia is generally asymptomatic. This disorder can be suspected when, despite adequate SIADH treatment, serum sodium remains low [58, 63].

In adults, the diagnosis of RO can be definitively performed through the evaluation of FEUa and by performing a water loading test (dose: 10–15 ml/kg) to suppress AVP secretion. RO will be confirmed by a normal FEUa (4–11%) and by the excretion of more than 80% of the water load within 4 h [42, 64, 65].

### Reset osmostat in children

To the best of our knowledge, RO pediatric cases are few and the clinical records are scarce. RO neonatal cases in the literature number 6 and all show an underlying congenital midline defect. Among them, one is a preterm baby with cleft lip and palate and normal karyotype, treated with oral sodium supplementation and fludrocortisone [65].

Since most RO cases affect patients with congenital midline defect, a midline craniofacial defect may be a clue for suspicion of RO. The pathogenesis of the syndrome could be related to the lesion involving baroreceptors or dependent on the dysfunction of the osmolality-sensitive hypothalamic neurons [66].

In a reported case, RO occurred in a child who showed translocation between chromosomes 13 and 10, in addition to a central nervous system midline defect [66]. In almost all cases, hyponatremia was persistent despite sodium administration and treatment with hydrocortisone and fludrocortisone [65, 67]. One more case was described in the infantile period, in a 6-month-old infant who showed failure to thrive as a unique symptom [67].

## Subtypes of SIADH: nephrogenic syndrome of inappropriate antidiuresis

NSIAD, also considered the hereditary form of SIADH [38] or type D SIADH [45], is a rare genetic X–linked disorder characterized by hyponatremia, serum hypoosmolality, euvolemia (no edemas), and inappropriately concentrated urine (> 200 mOsm/kg) with increased natriuresis and increased FEUa (> 11%). These clinical features are comparable to SIADH, but AVP levels allow the differential diagnosis, being low or undetectable in NSIAD [41, 43, 51, 68, 69]. Kidney, adrenal, and thyroid functions are normal [69, 70]. Characteristically, as happens in SIADH, normal FEUa (4–11%) can be observed after serum sodium correction [42].

In NSIAD, the kidneys’ inability to dilute urine is due to a mutation in the AVP receptor type 2 (V2R) gene (named *AVP2R*), resulting in its constitutive activity and excessive water reabsorption [51, 68]. More frequently, NSIAD onset occurs in neonates or infants, especially males, with resulting hemizygous mutation [51]. Heterozygous females can also be symptomatic, due to the selective inactivation of an X chromosome in the kidney tubular cells (lionization) [52].

Familial presentation can occur [52, 71], as well as sporadic cases. It is quite difficult to estimate NSIAD prevalence in adults and children, since its presentation can be mild or delayed and the condition may remain unrecognized. Adult patients can be asymptomatic or present sporadic episodes of hyponatremia; in some cases, diagnosis can be occasional after a water load test [41, 71]. NSIAD-associated hyponatremia can be acute, recurrent, or chronic, presenting severe degrees of severity, according to the disease’s entity and time of onset [70]. In early onset cases, severe acute hyponatremia can lead to neurological symptoms and potential neurological impairment [52, 71].

The V2R receptor may also be involved in loss-of-function mutations. Interestingly, a different amino acid substitution in position 137 of the *AVPR2* gene (the most frequently involved site in NSIAD), in particular the substitution of arginine with a histidine (R137H), can cause nephrogenic diabetes insipidus (NDI), showing opposite clinical features than NSIAD. In fact, NDI is characterized by the insensitivity of kidney collecting ducts to high levels of AVP, which leads to polyuria, polydipsia, hypernatremic dehydration, and excessively diluted urine [41].

### NSIAD in children

In 2005, Feldman et al. [44] first described NSIAD in two male infants (2.5 months and 3 months of age), and 3 years later, our group diagnosed and published the first neonatal case of the syndrome [69]. From the first description, many other cases of NSIAD have been reported; neonatal/infantile onset is more common, through acute hyponatremia accompanied by seizures, although such condition can persist in asymptomatic status until adulthood, being triggered by different stimuli, including infections or increased water intake [51, 69].

Our research group recently performed a detailed review on NSIAD presentation in neonates and infants. In the paper, an updated table on neonatal and infantile reported cases between 2005 and 2018 can be found, together with the specific genotype–phenotype correlation in all the available cases, age and symptoms at onset, performed treatments, and laboratory tests [41]. As reviewed, in infants, the most common trigger was represented by infections (especially respiratory syncytial virus (RSV)) and the passage from breastfeeding to formula milk or solid foods, accompanied by an increase in fluid intake [41].

To date, five kinds of point mutations of the *AVPR2* gene have been associated with NSIAD, all determining a single amino acid substitution associated with V2R gain of function, a G protein–coupled receptor expressed on the basolateral side of the principal cells in the collecting duct [41, 43, 44, 70]. More recently, mutations in the stimulatory Gα protein GNAS have been identified as other causes of NSIAD [72, 73].

Each of these reported mutations is associated with a different intracellular interaction pathway and clinical manifestations and, potentially, with a different pattern of drug response, as reviewed [41], underling the importance of genotype/phenotype correlation for therapeutic approach and clinical management.

### NSIAD diagnosis and treatment

NSIAD diagnosis can be confirmed by sequencing the *AVPR2* gene. If suspected, a water challenge test can also be useful to confirm NSIAD diagnosis, since affected patients are unable to eliminate a water load and develop hyponatremia, as happens in SIADH. Thus, a water challenge test is not conclusive in the differential diagnosis between SIADH and NSIAD [41, 44, 71]; this could be unsafe in newborns, especially in case of severe hyponatremia, and therefore is not recommended except in older and asymptomatic patients [71].

After diagnosis, screening of relatives is recommended, even if asymptomatic [70].

Regarding the treatment of hyponatremia, serum sodium level should be corrected, avoiding excessive rapidity, which could cause neurological damage [47, 52, 74].

Water restriction is a key point of treatment at all ages, even if, in neonates, an excessive reduction of fluid intake can interfere with adequate growth [53].

Urea oral administration is an effective, safe, and inexpensive treatment which generally leads to electrolyte normalization and can be continued as long as necessary; in some cases, it can be discontinued after weeks/months while, in other cases, it is continued for a long time [44, 51, 53, 69, 71, 74].

Our literature review points out that, under 1 year of age, water restriction, eventually followed by urea administration, has been the most common treatment for NSIAD-associated hyponatremia [41].

Although low/undetectable AVP circulating levels characterize NSIAD, with adequate treatment, a rise in AVP level can be observed, highlighting a residual regulation in AVP secretion when serum sodium, after correction, is not too low to inhibit AVP secretion [69].

In conclusion, NSIAD outcome and treatment depend on the time of onset and the degree of hyponatremia. NSIAD-correlated hyponatremia should be recognized and adequately treated to avoid the occurrence of neurological sequelae. The evolution of NSIAD and its response to the treatment is highly variable, as reported in the literature [41, 69].

## Discussion

Since CRSW syndrome and SIADH show some common findings, their differential diagnoses could be a difficult diagnostic challenge. Both disorders can be associated with a cerebral disease (including malformations, infections, tumors). As demonstrated in adults and in children and newborns, the ECV evaluation may be difficult and inadequate for a differential diagnosis between CRSW syndrome and SIADH. Patients with CRSW syndrome, due to increased natriuresis, initially show ECV depletion which can, in turn, rapidly normalize in euvolemia. AVP secretion occurs despite hyponatremia, following baroreceptor stimulation. Because of this, a CRSW syndrome–affected patient can appear euvolemic.

A simple algorithm can help clinicians to differentiate the two syndromes. Firstly, it is necessary to value the hyponatremia severity. A moderate to mild hyponatremia is usually seen in RO. Diluted urine with urine osmolality less than plasma osmolality with normal FEUa is also consistent with RO. In addition, RO does not respond to oral sodium supplementation or water restriction or fludrocortisone supplementation. Severe hyponatremia is typically found in CRSW syndrome and/or SIADH, and both conditions are characterized by hypouricemia, increased urine osmolality, and increased FEUa. Water restriction can normalize serum sodium, uricemia, and FEUa in SIADH. Conversely, a persistently elevated FEUa (FEUa > 11%) after hyponatremia correction identifies CRSW syndrome, even if the exact mechanism for this condition was not explained, to date. Correcting serum sodium is sufficient to normalize FEUa in SIADH, where damage involving the proximal tubule is obviously absent, but not in CRSW syndrome. Thus, it could suggest the presence of damage involving this region in CRSW syndrome, potentially involving a specific transporter regulating sodium excretion also.

Thus, even if natremia is corrected by exogenous sodium, the proximal tubule still eliminates sodium and uric acid, so the FEUa is increased as long as the kidney damage persists.

This hypothesis is supported by Lee et al. on tubular damage among adult patients [63]. Since FEUa is used in adult patients to differentiate the causes of hyponatremia, we hypothesized that it could be useful also in children over 1 year of life, together with other laboratory values and tests (Fig. 1) in discriminating among the forms in which clear hypovolemia is lacking.

Although a clear algorithm in adults is still available [75], we proposed one to be used in children, considering the inadequacy of clinical data. A prompt and opportune differential diagnosis is urgent, since treatment of CRSW syndrome and SIADH is different. In conclusion, the determination of serum uric acid and FEUa can distinguish CRSW syndrome from SIADH.

## Conclusions

Considering the inadequacy of clinical evaluation in distinguishing hypovolemic hyponatremia from euvolemic forms, FEUa was demonstrated as a reliable diagnostic tool, currently used in adult patients, in the discrimination between CRSW syndrome and SIADH. Through the above discussion, we propose FEUa use (combined with hyponatremia evaluation and urinary osmolality measurement) and its persistent alteration even after hyponatremia correction, among children over 1 year of life. CRSW syndrome is considered a self-limited disorder generally healing within a few months or years. The use of the proposed algorithm would also simplify CRSW syndrome diagnosis, allowing the diagnosis of cases that could be misunderstood and improving the evaluation of the kidney proximal tubule function.
